# Home Robot Interaction Based on EEG Motor Imagery and Visual Perception Fusion

**DOI:** 10.3390/s25175568

**Published:** 2025-09-06

**Authors:** Tie Hua Zhou, Dongsheng Li, Zhiwei Jian, Wei Ding, Ling Wang

**Affiliations:** 1 Department of Computer Science and Technology, School of Computer Science, Northeast Electric Power University, Jilin 132013, China; thzhou@neepu.edu.cn (T.H.Z.); 2202301048@neepu.edu.cn (D.L.); 2202400860@neepu.edu.cn (Z.J.); 2 Key Laboratory of Computing Power Network and Information Security, Ministry of Education, Shandong Computer Science Center (National Supercomputer Center in Jinan), Qilu University of Technology (Shandong Academy of Sciences), Jinan 250000, China; dingw@sdas.org; 3 Shandong Provincial Key Laboratory of Computer Networks, Shandong Fundamental Research Center for Computer Science, Jinan 250000, China

**Keywords:** motor imagery electroencephalogram (MI-EEG), feature fusion, scene recognition, home robot, human–robot interaction

## Abstract

Amid the intensification of demographic aging, home robots based on intelligent technology have shown great application potential in assisting the daily life of the elderly. This paper proposes a multimodal human–robot interaction system that integrates EEG signal analysis and visual perception, aiming to realize the perception ability of home robots on the intentions and environment of the elderly. Firstly, a channel selection strategy is employed to identify the most discriminative electrode channels based on Motor Imagery (MI) EEG signals; then, the signal representation ability is improved by combining Filter Bank co-Spatial Patterns (FBCSP), wavelet packet decomposition and nonlinear features, and one-to-many Support Vector Regression (SVR) is used to achieve four-class classification. Secondly, the YOLO v8 model is applied for identifying objects within indoor scenes. Subsequently, object confidence and spatial distribution are extracted, and scene recognition is performed using a Machine Learning technique. Finally, the EEG classification results are combined with the scene recognition results to establish the scene-intention correspondence, so as to realize the recognition of the intention-driven task types of the elderly in different home scenes. Performance evaluation reveals that the proposed method attains a recognition accuracy of 83.4%, which indicates that this method has good classification accuracy and practical application value in multimodal perception and human–robot collaborative interaction, and provides technical support for the development of smarter and more personalized home assistance robots.

## 1. Introduction

As the trend of population aging continues to intensify, the demand for home service robots in elderly people’s life assistance is growing. To achieve truly intelligent service response, the robot system must have the ability to perceive user intentions and environmental status, so as to complete context-related task reasoning and action execution. Recent advancements in Brain-Computer Interface (BCI) systems have opened new avenues for recognizing human intentions. Among them, Motor Imagery (MI) EEG signals, which allow for intention expression without muscular activity, have shown great promise in applications including neurorehabilitation and intelligent interactive systems [1].

Extracting motor intention from electroencephalograms (EEGs) has become a core issue in the field of Brain-Computer Interfaces (BCIs). Recent studies have demonstrated that EEG can effectively capture movement-related electrical activity in the brain and play a crucial role in decoding motor intention. Kim et al. demonstrated that EEG can be used to classify intended movement direction, providing a relevant precedent for the decoding method explored in this study [2]. In MI signal analysis, Common Spatial Pattern (CSP) is frequently utilized as a feature extraction technique due to its ability to maximize discriminative variance between classes [3]. Proposed by Ang et al., the Filter Bank Common Spatial Pattern (FBCSP) technique enhances CSP’s sensitivity to various frequency bands through the use of multi-band filtering, resulting in a marked improvement in classification accuracy [4]. Wavelet packet decomposition was introduced by Jahankhani et al. to segment EEG signals into various sub-bands, aiming to better capture their non-stationary properties for enhanced feature extraction [5]. Yuan et al. combined sample entropy with spatial features to effectively improve the separability of complex EEG signals [6]. Despite these advancements, the high-dimensional redundancy of MI-EEG signals poses significant challenges in computational efficiency and practical deployment in wearable systems. To improve signal processing efficiency and accuracy, various channel selection strategies have been introduced by scholars. Luo et al. sorted the channels based on Fisher Score and effectively retained the key channels [7]; Wang et al. used the maximum mutual information criterion to select feature channels and achieved a balance between information fidelity and dimensionality compression [8]. The channel optimization strategy developed by Jiang et al. was successfully applied to wearable BCI systems, significantly improving portability and actual deployment efficiency [9]. In summary, while various techniques such as CSP, FBCSP, and wavelet packet decomposition have advanced the field of EEG signal analysis, the challenges related to high-dimensional data and computational efficiency remain significant. Several channel selection strategies have been proposed to address these issues, demonstrating their potential to improve the practicality and efficiency of wearable BCI systems. However, further research is needed to integrate these techniques effectively and ensure robust performance in real-world applications.

On the other hand, scene recognition, as a key task of robot environmental perception, has also made great progress. A series of YOLO models [10] have become the mainstream method for real-time target detection with its end-to-end structure and efficient reasoning ability. YOLO v8 achieves better reasoning speed and structural flexibility while maintaining high detection accuracy. Laulkar et al. built a home image scene recognition system based on YOLO, and classified scenes such as kitchen and bedroom by detecting object categories and spatial distribution [11]. Zhang et al. proposed to use the target co-occurrence matrix to model the scene semantic relationship, which showed good generalization ability in complex environments [12].

Image feature extraction technology is also constantly evolving. AlexNet proposed by Krizhevsky et al. first achieved breakthrough image classification performance on ImageNet [13]. The VGG(Visual Geometry Group) network structure proposed by Simonyan and Zisserman provided a deeper feature representation [14]. Dosovitskiy et al. introduced the Transformer architecture to achieve unified modeling of images and language [15]. Zhu et al. proposed a framework that can fully perceive the co-occurrence relationship of labels in different scenarios while learning the visual representation of labels [16].

In multimodal interactive systems, the fusion of EEG and visual perception has become a research hotspot. An emotion recognition framework was introduced by Zhang et al., in which image and EEG information are combined through feature-level fusion using concatenation techniques [17]; Min et al. fused EEG and video features in fatigue detection, improving the detection accuracy while maintaining low latency [18]. In addition, Younis et al. constructed an emotion recognition model through a multi-sensor fusion strategy and verified that the integration of multi-source information improves classification performance [19]. Liu et al. proposed a hierarchical attention fusion framework to dynamically adjust the weight of modality contributions, enhancing the interpretability of multimodal inference tasks [20]. In terms of robot visual perception, Yang et al. constructed a multimodal semantic understanding framework to jointly reason about image, depth and speech inputs [21]; Duan et al. introduced an adaptive weighting mechanism to dynamically adjust the weight distribution of each modality according to task requirements [22]. EEG and video signals were further fused using deep neural networks by Choi et al., facilitating real-time user interaction and the prediction of intentions [23].

In recent studies, Senior et al. developed a method that combines semantic graphs with graph neural networks to model scene-task relationships in multimodal home interaction scenarios, offering promising results [24]. Additionally, Didolkar et al. examined the role of target center features in scene classification, demonstrating that training with diverse real-world images can enhance model generalization across unknown scenes [25]. Furthermore, the Places365 dataset has become a key benchmark for scene recognition, and Zhou et al.’s database has played a significant role in advancing the field [26]. Jocher et al.’s open-source Ultralytics YOLO framework also provides substantial support for scene detection algorithms, further enhancing the capabilities of YOLO-based systems [10].

Based on the research above, it is evident that while significant advancements have been made in EEG signal classification and visual scene recognition, there remains a gap in efficiently integrating user intentions and environmental semantics for enabling intelligent home robots to respond in real-world multi-task scenarios. In response to this gap, this work introduces a multimodal interaction framework, incorporating optimized EEG channel selection for MI classification and YOLO v8-based scene recognition, capable of reliably distinguishing and generating commands for 12 different tasks. Each scene corresponds to 4 specific tasks, so that the robot can flexibly respond to user intentions in different scenarios to achieve “intention-scene-task” linkage recognition. The main contributions of this paper are as follows:

1. A multi-frequency band and multi-feature fusion framework for MI-EEG classification is proposed, which integrates FBCSP, wavelet packet decomposition, and sample entropy to improve intention recognition accuracy.
2. A YOLO v8-based target detection and scene classification model for home environments is constructed, supporting efficient recognition of scene semantics.
3. A fusion strategy for EEG intentions and visual scenes is designed, establishing the “intention-scene-task” mapping to generate multimodal interactive task instructions.

This paper is organized as follows: Section 2 describes the materials and methods, Section 2.2 introduces the motor imagery EEG classification method based on channel selection and feature fusion (CSFF Model), Section 2.3 describes scene recognition based on object detection result feature construction (ODFC Model), Section 2.4 introduces decision-level integration and robot command mapping, Section 3.2 shows the channel selection results and evaluation results of the CSFF Model, Section 3.3 shows the evaluation results of the ODFC Model, Section 3.4 evaluates the multimodal human–robot interaction model, Section 4 describes the direction of future work, and Section 5 summarizes the study and results.

## 2. Materials and Methods

### 2.1. Motivation

**Figure 1:**
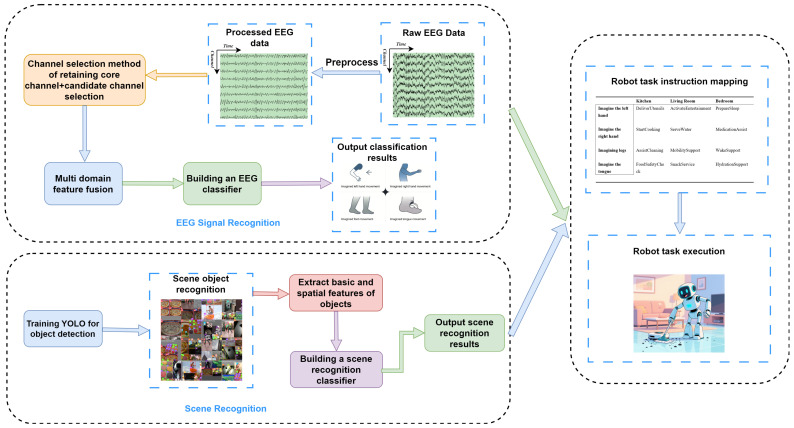
Overall framework.

Most existing EEG recognition relies on full-channel data, which limits the actual deployment of the system. Improving the efficiency of EEG decoding and the portability of the system necessitates a robust channel selection mechanism that retains informative channels, along with feature extraction methods capable of sustaining recognition performance under reduced-channel conditions. On the other hand, although the visual scene recognition method has good perception ability, it is separated from the user’s intention and cannot achieve a demand-based scene response. Most current multimodal systems are shallowly fused and lack unified modeling of intent and environment. Therefore, this paper aims to compress the EEG input dimension through channel screening, combine multi-feature fusion to improve classification performance, introduce YOLO v8 (Ultralytics, Los Angeles, CA, USA) [10] for visual scene understanding, and jointly model the two so that the robot can flexibly respond to user intentions in different scenarios and perform operations matching the scene to achieve task-driven human–robot collaborative interaction. The overall framework of this study is illustrated in Figure 1.

### 2.2. Motor Imagery EEG Classification Method Based on Channel Selection and Feature Fusion (CSFF Model)

**Figure 2:**
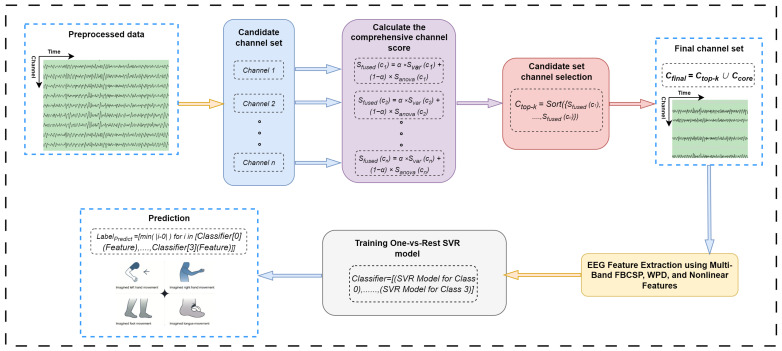
Channel selection flowchart, where channeln represents the set of channels after removing the core channels, $S_{var}$ represents the Discriminative Variance Score, $S_{anova}$ represents the Band Power Significance Score, $C_{final}$ represents the set of channels that are finally selected, and $C_{core}$ represents the retained core channel. SVRModelforClassn represents the classification model constructed for the *n*-th class of EEG signals, and *i* represents the SVR prediction value.

A classification method for motor imagery EEG signals is introduced, incorporating both channel selection and feature fusion techniques. Discriminative channels are first selected based on their performance in key frequency bands. Then, a high-dimensional multimodal feature set is constructed by integrating time–frequency-, spatial-, and entropy-based features, followed by feature fusion to enhance representation. Finally, Support Vector Regression is employed to accurately classify four motor imagery tasks. See Figure 2 for details.

#### 2.2.1. Datasets and Preprocessing

The BCI Competition IV Dataset 2a [27] contains EEG data from 9 subjects performing four distinct motor imagery tasks. Data were acquired using 22 electrodes with a 250 Hz sampling rate. Each subject underwent multiple runs, completing a total of approximately 576 trials, with each trial lasting about 4 s. The BCI Competition III Dataset 3a [28] is also used for motor imagery tasks. This data includes EEG data from three subjects covering four categories of motor imagery (left hand, right hand, foot, and tongue). It was recorded using 60 channels, with a sampling rate of 250 Hz, a 1–50 Hz bandpass, and a power frequency notch. Each subject underwent at least six runs, each consisting of 40 trials, for a total of approximately 60 trials per category. Each trial lasted approximately 7 s: 0–2 s of rest; at t = 3 s, the arrow cue was given and imagery began, continuing until t = 7 s.

During EEG signal preprocessing in this study, a bandpass filter (frequency range 8 to 32 Hz) was first applied, using a fourth-order Butterworth filter to effectively remove low-frequency drift and high-frequency noise, while retaining frequency bands associated with motor imagery. This frequency band selection was based on the characteristics of movement-related electrical activity. Independent component analysis (ICA) was used to remove eye movement and electromyographic artifacts. ICA decomposition and the manual removal of independent components related to eye movement and electromyography further improved the purity of the EEG signal. Furthermore, baseline correction was performed to eliminate the pre-stimulus baseline drift. Specifically, a baseline window from −200 ms to 0 ms was used, and the mean of this window was subtracted to remove baseline drift and ensure signal stability. For analysis, the preprocessed EEG signal is segmented into fixed-length 2 s time windows, with a 0.04 s interval between each window. This results in 26 distinct time windows for the EEG data between 1 and 4 s. To improve classification accuracy, a multimodal feature extraction method is employed. This method combines spatial discrimination, a time–frequency energy structure, and nonlinear dynamic complexity, specifically applied to EEG signals from motor imagery tasks. By integrating frequency–bandwidth cospatial patterns (FBCSPs), wavelet packet decomposition, and nonlinear features, this method effectively captures differences in neural activity across different brain regions, frequency bands, and structural levels.

#### 2.2.2. Channel Selection Method

In BCI systems, high-density EEG provides rich information but also introduces redundancy, noise, and computational overhead, potentially reducing classification accuracy and stability. In response to this challenge, we design a channel selection method that leverages both physiological priors and statistical data analysis: a core channel retention and candidate channel dynamic selection method, balancing spatial filter stability and channel discriminability. The approach involves the following key steps:

- Core Channel Retention;

The motor imagery task is highly dependent on the activity of the Primary Motor Cortex (PMC), which is mapped on the scalp EEG mainly to $C_{3}$ (left-handed area), $C_{z}$ (central area) and $C_{4}$ (right-handed area). To maintain the stability and physiological interpretability of spatial feature extraction algorithms (e.g., CSP), the following core channels are fixedly retained in this paper: $C_{\mathrm{core}}=\left\{C_{3},C_{z},C_{4}\right\}$. Let the original set of channels be $C_{\mathrm{all}}$, then the set of candidate channels is

(1) $$C_{\mathrm{cand}}=C_{\mathrm{all}}\setminus C_{\mathrm{core}}$$

The symbol “\” represents subtraction. Next, the candidate channels are evaluated, channels with high channel scores are selected, and the final set of channels comprises the core channels and the selected channels.

- Candidate Channel Scoring Mechanism;

In the motor imagery EEG classification, channel discriminability varies across motion classes. To reduce redundancy while preserving key information, this paper introduces two statistical scoring metrics to quantify signal distribution and class separability, guiding effective channel selection.

Discriminative Variance Score: EEG signals exhibit task-specific spatial patterns in MI tasks. Channels with high variance in specific tasks and moderate variance across all trials indicate strong selectivity and class discrimination. Such characteristics enable efficient channel screening. The evaluation metric is defined as follows:

(2) $$S_{\mathrm{var}}\left(c_{i}\right)=\frac{1}{N}\sum_{j=1}^{N}\left(\frac{\sigma_{ij}^{2}}{\sigma_{i}^{2}+\epsilon }\right)$$

In the formula, $\sigma_{ij}^{2}$ represents the signal variance of channel $c_{i}$ in the *j* category, which is used to measure the fluctuation of the channel’s signal in a particular category; $\sigma_{i}^{2}$ is the total variance of channel $c_{i}$ across all trials, reflecting the overall level of fluctuation of the channel; *N* is the number of categories, which in this task is *N = 4* (4 categories of EEG-related motor imagery); and ϵ is a smoothing factor, which is designed to avoid having a zero in the denominator and to ensure that the formula calculations are reasonable.

Band Power Significance Score: ERD/ERS (Event-Related Desynchronization/Event-Related Synchronization) responses in the μ (8–13 Hz) and β (13–30 Hz) bands are typically evoked by motor imagery tasks involving the left hand, right hand, foot, and tongue, providing substantial class-discriminative information. Channels with significant power differences across tasks are more informative. To quantify this, we introduce the ANOVA Power Score [29], Specifically, for each channel, and the mean square power is computed after filtering, grouped by task labels, and evaluated using one-way ANOVA. The steps involved are presented as follows.

Power feature extraction: Suppose the signal of a certain channel $c_{i}$ in the *t*-th trial is $x_{i}^{\left(t\right)}\in R^{T}$. The power of this trial is shown in Formula (3):

(3) $$P_{t}^{\left(i\right)}=\frac{1}{T}\sum_{k=1}^{T}{\left(x_{t}^{\left(i\right)}\left[k\right]\right)}^{2}$$

where *T* represents the number of sampling points in the *trial*, and $x_{t}^{\left(i\right)}\left[k\right]$ is the signal value of the *k*-th sampling point. This power feature essentially reflects the energy level of the channel in the current trial.

Significance testing method: The power features are divided into four groups according to the category labels of *trial*:

(4) $$\left\{P_{t}^{\left(i\right)}|y_{t}=j\right\},\;j\in \left\{0,1,2,3\right\}$$

where $P_{t}^{\left(i\right)}$ represents power, and j represents the category of motor imagery EEG (left hand, right hand, foot, and tongue). Analysis of variance (ANOVA) is conducted to determine if there is a significant difference in the means of the four power samples, under the null hypothesis

(5) $$H_{0}:\mu_{0}=\mu_{1}=\mu_{2}=\mu_{3}$$

If $H_{0}$ is rejected, it means that the channel has a statistically significant power distribution difference between categories and has discriminatory power. In order to enhance the discriminatory nature of the scoring and so that a larger score value indicates a more important channel, the negative logarithm of the *p*-value is used as the score:

(6) $$S_{\mathrm{anova}}\left(c_{i}\right)=-\log_{10}\left(p{-\mathrm{value}}_{i}\right)$$

where *p*-value_*i*_ represents the significance probability of the ANOVA test result for channel *$c_{i}$*. Taking its logarithm can stretch the value range and avoid the problem of insensitivity to small numbers.

Fused Channel Score and Channel Selection: The discriminative variance score and band power significance score assess channel discriminability from complementary perspectives—signal volatility and frequency band energy. To better capture their combined strengths, we design a fusion strategy to compute a composite score for more comprehensive channel importance evaluation.

To eliminate the influence of the measurement scales and the differences in value ranges of different evaluation indicators, first, for the initial scores $S_{\mathrm{var}}\left(c_{i}\right)$ and $S_{\mathrm{anova}}\left(c_{i}\right)$, the normalization form is as follows:

(7) $${\tilde{S}}_{\mathrm{var}}\left(c_{i}\right)=\frac{S_{\mathrm{var}}\left(c_{i}\right)-\min\left(S_{\mathrm{var}}\right)}{\max\left(S_{\mathrm{var}}\right)-\min\left(S_{\mathrm{var}}\right)}$$

(8) $${\tilde{S}}_{\mathrm{anova}}\left(c_{i}\right)=\frac{S_{\mathrm{anova}}\left(c_{i}\right)-\min\left(S_{\mathrm{anova}}\right)}{\max\left(S_{\mathrm{anova}}\right)-\min\left(S_{\mathrm{anova}}\right)}$$

The normalized scores all fall within the interval [0,1], which facilitates fusion. The final fusion score is calculated as

(9) $$S_{\mathrm{fused}}\left(c_{i}\right)=\alpha \cdot {\tilde{S}}_{\mathrm{var}}\left(c_{i}\right)+\left(1-\alpha \right)\cdot {\tilde{S}}_{\mathrm{anova}}\left(c_{i}\right)$$

where α∈[0,1] is an adjustable weight parameter representing the proportion of the variance score in the final score. In this paper, an equal-weight strategy is adopted, i.e., α=0.5, indicating that the two types of indicators are equally important.

According to the fused score $S_{\mathrm{fused}}\left(c_{i}\right)$, all candidate channels are ordered by descending importance, and the top *K* are selected as the optimal channel subset for feature extraction and classification tasks.

#### 2.2.3. EEG Feature Extraction

Multi-band filtering and FBCSP spatial feature extraction: In the motor imagery task, different motor tasks will activate different areas of the brain, and these areas show different activity patterns in different frequency bands (such as α and β). The FBCSP (Filter Bank Co-spatial Pattern) method can effectively extract the spatial covariance difference between each frequency band and the task through multi-band filtering and spatial projection [30], thereby improving the accuracy of classification.

The Common Spatial Pattern (CSP) aims to find the best spatial filter $W\in R^{m\times k}$ (m represents the number of channels, and k denotes the feature dimension retained after filtering) to maximize the covariance difference between the two types of data. Let the covariance matrices of the two types of signals be $C_{1}$ and $C_{2}$. Then the optimization goal is the following:

(10) $$\arg\max_{W}\frac{\mathrm{tr}(W^{T}C_{1}W)}{\mathrm{tr}(W^{T}\left(C_{1}+C_{2}\right)W)}$$

(11) $$W^{T}\left(C_{1}+C_{2}\right)W=I$$

The obtained filter *W* projects the signal to a new space $Z=W^{T}X_{b}$, where the variance distribution of the projected components reflects the class discriminability. The steps of FBCSP feature extraction are as follows: First, the EEG signal is divided into 11 frequency bands (e.g., 8–12 Hz, 10–14 Hz, …, 28–32 Hz). For each frequency band *b*, the covariance matrices $C_{1b}$ and $C_{2b}$ corresponding to the two data are calculated. On this basis, the CSP filter $W_{b}$ is solved, and the frequency band signal $X_{b}$ is projected into a new space to obtain $Z_{b}=W_{b}^{T}X_{b}$; then, by calculating the logarithm of the ratio of the variance of each component to the total variance, the first most discriminative component $Z_{b1},Z_{b2},Z_{b3},Z_{b4}$ is selected after projection. The formula is as follows:

(12) $$f_{i}=\log\left(\frac{\mathrm{var}(Z_{i})}{\sum_{j=1}^{k}\mathrm{var}\left(Z_{j}\right)}\right)$$

where $Z_{i}$ is the *i*th CSP component, and *k* represents the number of selected channels. After extracting features from a single frequency band, the features from the 11 frequency bands are concatenated in sequence to generate the final feature vector $F_{FBCSP}$ with 44 dimensions.

Wavelet Packet Decomposition (WPD) Time–Frequency Domain Feature Extraction: In the motor imagery task, EEG energy in the α and β bands exhibits Event-Related Desynchronization/Synchronization (ERD/ERS) effects, and the temporal patterns vary from individual to individual. Unlike Fourier transform, WPD captures frequency and temporal dynamics through multi-level decomposition, effectively extracting the non-stationary features of EEG [31]. WPD recursively divides the signal into low-frequency and high-frequency sub-bands, forming a tree structure, in which each node represents a specific frequency band for refined time–frequency analysis. Taking the 5-layer decomposition as an example, the first layer decomposes the original signal into low-frequency sub-band $A_{1}$ and high-frequency sub-band $D_{1}$; the second layer further decomposes $A_{1}$ into ${\mathrm{AA}}_{2}$ and ${\mathrm{AD}}_{2}$ and divides $D_{1}$ into ${\mathrm{DA}}_{2}$ and ${\mathrm{DD}}_{2}$, and so on. After each layer of decomposition, the number of sub-bands increases exponentially. The nth layer contains $2^{n}$ nodes, each of which corresponds to a specific frequency range. This paper performs a 5-layer WPD decomposition on the signal on each channel and selects 6 key sub-band nodes for feature extraction. The features of frequency bands related to motion imaging tasks (such as 8–32 Hz) can be extracted. For each selected child node b, the signal strength of the frequency band is quantified by calculating the energy feature. The calculation formula is

(13) $$E_{b}=\sum_{n=1}^{N}{\left|x_{b}\left(n\right)\right|}^{2}$$

where $x_{b}\left(n\right)$ represents the signal value of node *b* at the *n*-th sampling point, and  *N* represents the coefficient sequence length for current node. Essentially, this formula is the sum of the squares of the signal amplitudes of the node, directly reflecting the energy magnitude of the signal in this frequency band. For example, if a node corresponds to the α frequency band and an ERD phenomenon occurs in this frequency band during a motor imagery task, its energy $E_{b}$ will be significantly reduced. The obtained 90-dimensional feature is marked as $F_{WPD}$.

Nonlinear Feature Extraction: EEG signals are essentially the output of complex nonlinear systems, containing nonlinear dynamic characteristics such as chaotic behavior and self-similarity. Traditional linear features are limited by linear assumptions and are difficult to characterize complex dynamic patterns of signals. In order to break through the limitations of linear analysis, it is necessary to introduce nonlinear features such as sample entropy, spectral entropy, and fractal dimension. By mining the complex structure and dynamic laws of signals, the accuracy of EEG signal classification can be improved, providing richer and more effective information for EEG signal processing and analysis.

Sample Entropy (SampEn): Sample entropy quantifies the complexity and randomness within a time series, serving as an indicator of the signal’s intrinsic regularity. During calculation, by comparing the number of matching pairs of sequences under different embedding dimensions, the complexity indicator is derived:

(14) $$\mathrm{SampEn}\left(m,r,N\right)=-\ln\left(\frac{A}{B}\right)$$

In the formula, *m* denotes the embedding dimension, which controls the complexity of the sequence reconstruction space; *r* is the tolerance radius, which defines the threshold for sequence similarity matching; *N* denotes the length of the signal, representing the amount of analysis data; *A* and *B* correspond to the number of sequence matching pairs under the embedding dimensions of m+1 and *m*, respectively. The obtained features are expressed as $F_{\mathrm{Sa}}$. The lower the sample entropy value, the more significant the regularity of the signal; the higher the value, the stronger the randomness and irregularity of the signal.

Spectral Entropy (SpecEn): Spectral entropy quantifies the uncertainty in a signal’s energy distribution within the frequency domain, reflecting the extent of energy dispersion across the spectrum:

(15) $$\mathrm{SpecEn}=-\sum_{i=1}^{n}p_{i}\log_{2}\left(p_{i}\right)$$

where $p_{i}$ is the normalized spectral power, satisfying $\sum_{i=1}^{n}p_{i}=1$ (where *n* is the number of spectral bins).The obtained features are expressed as $F_{\mathrm{Sp}}$. An increase in spectral entropy corresponds to a more dispersed energy distribution across the frequency domain, which in turn indicates a higher complexity in the signal’s frequency-domain characteristics.

Fractal Dimension (FD): The fractal dimension quantifies the complexity or roughness of a signal, depicting the degree of change in the signal’s morphology:

(16) $$FD=\frac{\log(L(k))}{\log(1/k)}$$

In the formula, L(k) is the average length of the signal at step *k*, reflecting the signal’s morphology at different scales; *k* is a variable step length, covering the local and global features of the signal through multi-scale analysis. The obtained features are expressed as $F_{\mathrm{Fd}}$. The higher the fractal dimension, the more complex the change in the signal’s morphology, and the more prominent the nonlinear features. The final 45-dimensional nonlinear features are $F_{NL}=\left[F_{\mathrm{Sa}},F_{\mathrm{Sp}},F_{\mathrm{Fd}}\right]$.

The obtained features are concatenated and dimensionality reduced by the PCA (Principal Component Analysis) algorithm that maps high-dimensional features to a set of linearly independent principal components, and sorts from large to small according to the variance contribution, and finally implements standardization. Finally, Support Vector Regression (SVR) is used to realize the four-category recognition of EEG signals. A SVR model is trained for each category through a one-to-many strategy, and the label of the target category is set to 1 and the labels of other categories are set to 0. During prediction, the four models output the “closeness” of each sample to each category, and finally select the category with the predicted value closest to 1 as the classification result of the sample. The overall algorithm steps are as in Algorithm 1.

### 2.3. Scene Recognition Based on Object Detection Result Feature Construction (ODFC Model)

To more clearly illustrate the design decisions of the proposed system, this study used a home environment representative of the daily lives of older adults. The living room, bedroom, and kitchen were chosen as the primary control environments for the system based on their frequent needs in the lives of older adults. The living room is a core area for daily leisure and social activities, the bedroom serves as a place for rest and recovery, and the kitchen is closely associated with diet and health management. The combination of four motor imagery categories (left hand, right hand, legs, and tongue) with these three environments aims to achieve task control that adapts to the diverse needs of older adults. For example, motor imagery of the left hand can be used to control lights, the right hand can be used to operate a TV or open and close curtains, and motor imagery of the legs and tongue can be used for tasks such as walking or interacting with robots through voice. Through this design, we hope to help older adults achieve greater independence and autonomy in their daily lives, improving their quality of life.

**Figure 3:**
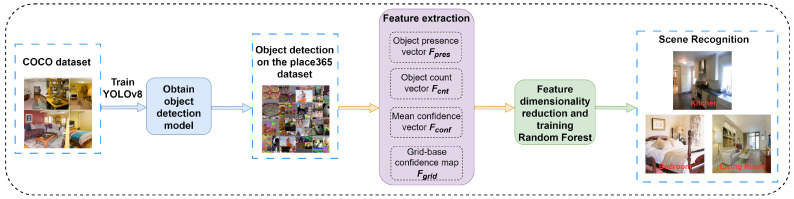
Scene Recognition flowchart.

In this study, the YOLO v8 model was trained to detect target objects in home scenes, and features such as object confidence and spatial distribution were extracted during the process. These features were then used to achieve automatic recognition of the scene, and classification and recognition were performed through machine learning methods. The relevant process is shown in Figure 3.
**Algorithm 1:** EEG channel selection, feature extraction, and classification.
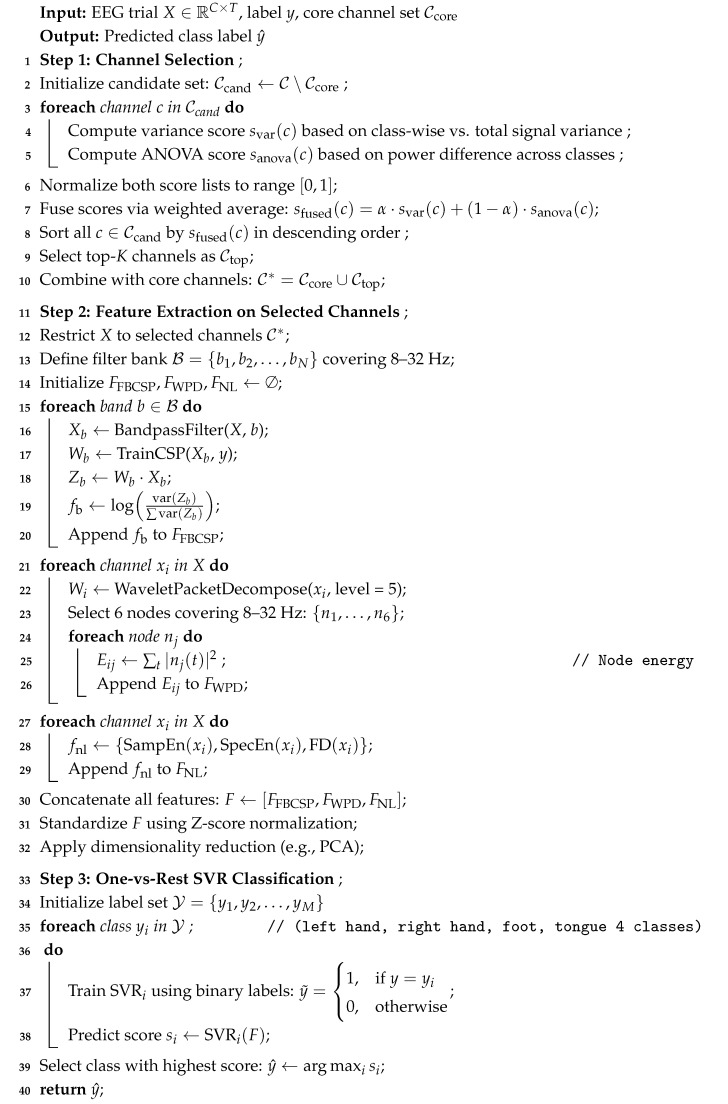


#### 2.3.1. Object Detection Model

In this study, YOLO v8 is selected as the detection model for its superior detection accuracy and rapid inference speed, which is particularly suitable for robot platforms with limited hardware resources. It has a lightweight architecture, end-to-end design, and excellent deployment support, making it the first choice for robot environment target detection.

We use the COCO (Common Objects in Context) dataset [32] to train the object detection model YOLO v8. The COCO dataset is one of the most representative public datasets for image recognition and object detection. It contains more than 330,000 images, of which more than 200,000 are annotated images, covering 80 general target categories, which are widely distributed in daily life scenes, such as TV, sofa, bed, furniture, electrical appliances, etc. The diversity and complexity of this dataset provide a good foundation for the generalization ability of the model in actual complex scenes.

For data partitioning, we adhere to the official COCO standard. The training set (train2017) consists of approximately 118,000 images, the validation set (val2017) includes about 5000 images, and the test set (test-dev) contains around 20,000 unannotated images, which are used to evaluate the model’s detection performance. The model is trained on this dataset to learn and classify a variety of target categories.

During training, images were resized to 640 × 640 pixels, with a batch size of 16. The model was trained for 100 epochs, using the SGD (mini-batch Stochastic Gradient Descent) [33] optimizer and an initial learning rate of 0.01. To enhance generalization, the training process incorporated Mosaic and MixUp data augmentation techniques [34], and a combined loss function, including bounding box regression, classification, and confidence losses, was used for optimization.

#### 2.3.2. Feature Extraction and Model Construction for Scene Recognition

- Feature Construction;

To achieve efficient and interpretable indoor scene recognition suitable for robot platforms, we propose a lightweight feature construction method based on the output of object detection. Specifically, four types of features are extracted from the detection results of the YOLO v8 model, namely: Object Presence, Object Count, Mean Confidence, and Grid-based Confidence Map. This method simultaneously fuses semantic, statistical, and spatial layout information, and has good real-time performance and scene discrimination ability.

**Object Presence Vector:** For each image, a binary vector $f_{\mathrm{pres}}\in {\left\{0,1\right\}}^{C}$ is constructed, where *C* represents the total number of target categories. If a target of a certain category is detected in the image, the corresponding position is marked as 1; otherwise, it is 0. This feature can directly express which types of objects are contained in the scene and is the most basic semantic description.

**Object Count Vector:** We further construct an integer vector $f_{\mathrm{cnt}}\in N^{C}$, where the *c*-th element represents the number of instances of category *c* detected in the current image. This feature can effectively reflect the density and type distribution differences of objects in the scene, and is helpful for distinguishing more complex scenes such as kitchens from relatively simple scenes such as bedrooms.

**Mean Confidence Score Vector:** For each object category *c*, we calculate the average confidence of all instances of that category of targets, defined as follows:

(17) $$f_{\mathrm{conf}}^{\left(c\right)}=\frac{1}{N_{c}}\sum_{i=1}^{N_{c}}s_{i}\;\left(N_{c}>0\right)$$

where $s_{i}$ denotes the confidence of the *i*-th target, and $N_{c}$ is the number of targets of category *c*; if $N_{c}=0$, it is recorded as 0. This feature measures the “degree of trust” of the model in various objects and can assist in suppressing interference caused by misdetections.

**Grid-based Confidence Map:** To characterize the spatial distribution pattern of objects in an image, we divide the image into N×N uniform grids (e.g., N=3), and count the confidence of each category of targets within each grid cell. Let $I_{c,i,j}$ represent the set of indices of targets of category *c* located in grid cell (i,j). The grid statistic is defined as

(18) $$f_{\mathrm{grid}}^{\left(c,i,j\right)}=\sum_{k\in I_{c,i,j}}s_{k}$$

The grid map of each category of targets is flattened into a vector $f_{\mathrm{grid}}^{\left(c\right)}\in R^{N^{2}}$, and then all categories are concatenated to obtain the total feature vector:

(19) $$f_{\mathrm{grid}}=\left[f_{\mathrm{grid}}^{\left(1\right)},f_{\mathrm{grid}}^{\left(2\right)},\ldots ,f_{\mathrm{grid}}^{\left(C\right)}\right]\in R^{C\times N^{2}}$$

By utilizing this feature, the model’s capacity to understand the spatial layout of common objects in the scene is enhanced (e.g., beds are usually found in the lower central portion of the image).

We concatenate the above four types of features to form the final scene representation vector $f_{\mathrm{scene}}$ at the image/frame level: $f_{\mathrm{scene}}=\left[f_{\mathrm{pres}},f_{\mathrm{cnt}},f_{\mathrm{conf}},f_{\mathrm{grid}}\right]\in R^{3C+C\cdot N^{2}}$. To reduce feature redundancy and enhance generalization, we apply Principal Component Analysis (PCA) to compress the high-dimensional scene feature vector into a lower-dimensional representation while retaining most of the variance. The resulting compact features are then fed into a Random Forest classifier to perform the final scene classification. The classifier outputs one of the predefined scene categories (i.e., kitchen, living room, or bedroom), which serves as the contextual basis for subsequent robotic decision-making. The detailed steps of the scene recognition and classification algorithm are shown in Algorithm 2.
**Algorithm 2:** Scene recognition and classification.
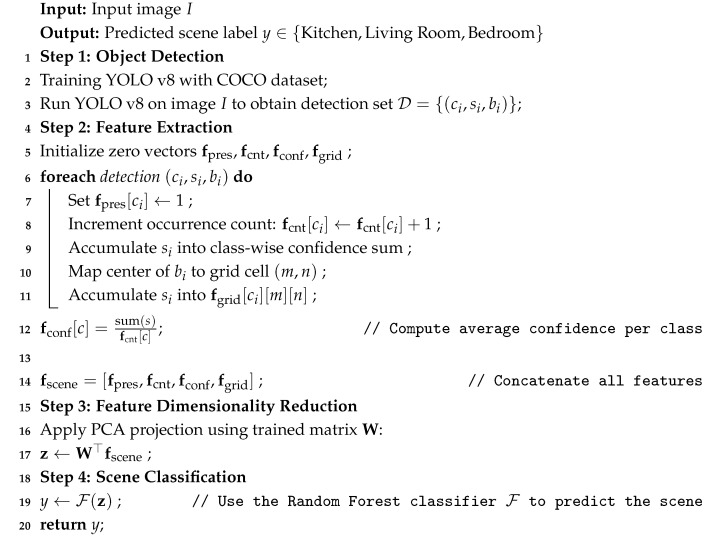


### 2.4. Decision-Level Integration and Robot Command Mapping

The mapping between motor imagery (MI) EEG signals and scenario tasks is designed based on the correlation between the specific task requirements of each scenario and the MI-EEG categories. In daily life, the left hand is typically used for delicate manipulations, such as delivering objects and operating kitchen equipment. The right hand is typically the dominant hand, so in many tasks, right-hand movements assume important roles. For example, in the living room, the right hand might be responsible for pouring water or offering other items. Tongue motor imagery can reflect some of the specific needs of the elderly in daily life. For example, in a kitchen scenario, tongue motor imagery can be used to determine whether food is suitable for consumption, and the EEG signal simulating tongue movement can trigger a food temperature check task. Leg motor imagery is primarily used to simulate walking, standing, or other mobility-related tasks. By recognizing the motor intention and scenario, the system can perform specific tasks that are highly correlated with these intentions and scenarios.

This scheme constructs a prediction distribution for 12 types of joint tasks by fusing the posterior probabilities of an independently trained EEG classifier and a scene recognition model. Specifically, assuming that the EEG classifier outputs $P_{\mathrm{MI}}\left(m_{i}\right)$ and the scene recognizer outputs $P_{\mathrm{scene}}\left(s_{j}\right)$, the joint probability for each task $t_{i,j}$ is calculated as follows:

(20) $$P_{\mathrm{task}}\left(t_{i,j}\right)=P_{\mathrm{MI}}\left(m_{i}\right)\cdot P_{\mathrm{scene}}\left(s_{j}\right)$$

The final prediction result is

(21) $$\hat{t}=\arg\max_{i,j}P_{\mathrm{task}}\left(t_{i,j}\right)$$

In order to realize the intelligent task scheduling and personalized response of the robot, this paper designs a command mapping method based on the joint decision-making of motor imagery EEG and scene recognition. Specifically, the EEG signal is decoded into four motor imagery categories (left hand, right hand, foot, and tongue) through the classification model, which represents the basic action instructions of the user’s intention; at the same time, the visual module identifies the current indoor scene type (kitchen, living room, and bedroom). By combining and mapping the two, each combination corresponds to a specific robot action instruction or service behavior. The specific mapping relationship is shown in Table 1.

## 3. Results

The computer used in this experiment is equipped with an AMD5000 series processor and an RTX3060 graphics card. Four datasets are used in the experiment. COCO is a widely used image recognition and detection dataset containing more than 200,000 images and 80 object categories. This study uses the COCO dataset to train the YOLO v8 target detection model. Places365 [35] is a large scene recognition dataset containing 365 scene categories and more than 1.8 million images. This study selects three typical home scene categories, namely “kitchen” “living room” and “bedroom”, to train the scene classifier. The BCI Competition IV Dataset 2a and BCI Competition III Dataset 3 are used to support the EEG part of the research. For detailed information about the datasets, please refer to Section 2.2.1 and Section 2.3.1 The ratio of each dataset used for training and testing is 7:3.

### 3.1. Parameter Evaluation

In this study, we conducted parameter selection for the filter frequency range, wavelet packet decomposition level, and time window length. We employed a stepwise optimization strategy to determine the optimal configuration for each parameter and used average accuracy as the evaluation metric. Specifically, starting from given initial values, we evaluated the effect of each parameter individually and gradually adjusted until reaching the optimal value. When adjusting one parameter, the other parameters were maintained at their default values or the optimal values determined in the previous step. The filter frequency range was initially set to 4–40 Hz. The wavelet packet decomposition level was initially set to 3 levels. The time window length was initially set to 2 s, with a time window interval of 0.04 s.

Firstly, we selected the frequency range of the filter. When the initial setting was 4–40 Hz, the classification accuracy was 73.2%. Next, we tested two other frequency band settings: 8–32 Hz and 8–30 Hz. The test results showed that the 8–32 Hz band provided the best classification accuracy of 74.5%. Therefore, 8–32 Hz was selected as the optimal frequency range and used as the default setting for subsequent steps. The comparison results can be found in Table 2.

After determining the optimal filter frequency range (8–32 Hz), we proceeded to optimize the number of layers in the wavelet packet decomposition. Under the default 2 s time window and optimal frequency band (8–32 Hz) conditions, we evaluated different numbers of wavelet packet decomposition layers (3, 4, and 5). The test results indicated that the 5-layer wavelet packet decomposition achieved the highest classification accuracy of 76.8%, compared to 74.5% and 75.2% for the 3-layer and 4-layer decompositions, respectively. Therefore, the 5-layer wavelet packet decomposition was selected as the optimal configuration. The comparison results can be found in Table 3.

Finally, we optimized the length of the time window. Under the conditions of the optimal frequency band (8–32 Hz) and the number of wavelet packet decomposition levels (5 levels), we tested different time window lengths (1 s, 2 s, and 3 s). The results showed that a 2 s time window provided the best balance, with a classification accuracy of 76.8%. Although the 3 s time window achieved an accuracy of 77.4%, considering the potential introduction of redundant information, the 2 s time window was selected as the optimal time window length. It provided a good balance between efficiency and accuracy. The comparison results can be found in Table 4.

### 3.2. CSFF Model Evaluation

#### 3.2.1. Channel Selection Results

**Figure 4:**
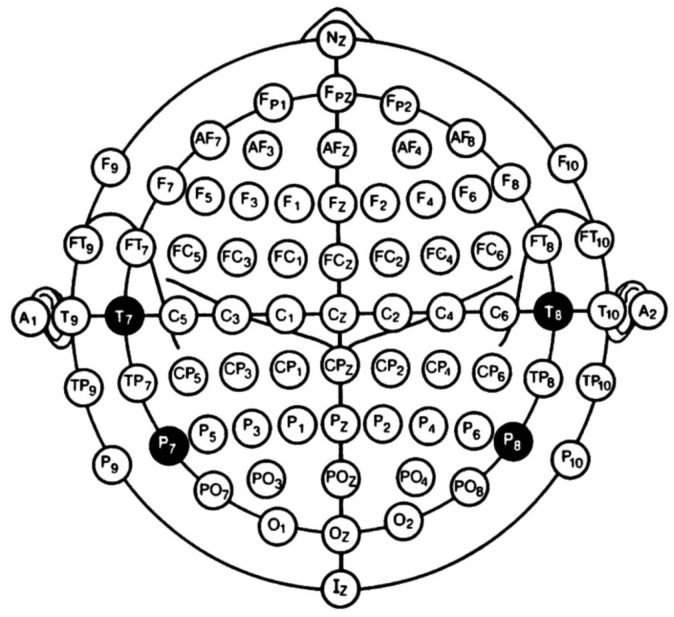
The 10-10 system electrode lead diagram.

To enhance the model’s universality and portability, the channel selection experiment was based on the international 10-10 system electrode nomenclature [36], and the original electrode numbers in the dataset were uniformly mapped to standard channel names. The 10-10 system electrode lead diagram is shown in Figure 4. Three core channels that are highly related to motor imagery were retained during the selection process: C3, C4, and Cz. On this basis, the total number of channels was set to 16. That is, from the remaining channels except the core channels, they were sorted according to the preset scoring mechanism, and the 13 candidate channels with the highest scores were selected. Table 5 displays the channel selection results for Dataset 2a, and Table 6 presents the corresponding results for Dataset 3a.

Two channel configurations, namely the full channel setup and a core channel combination (C3, C4, Cz), are compared with the CSFF-Channel Selection method introduced in this study. The specific experimental results, obtained using the proposed feature extraction and classification algorithm, are presented in Table 7. The results show that although the number of channels is significantly reduced, the accuracy difference between CSFF-Channel Selection and full-channel configuration is approximately 3%, thereby highlighting its advantages in achieving a balance between model performance and system portability.

#### 3.2.2. CSFF Model Test

**Figure 5:**
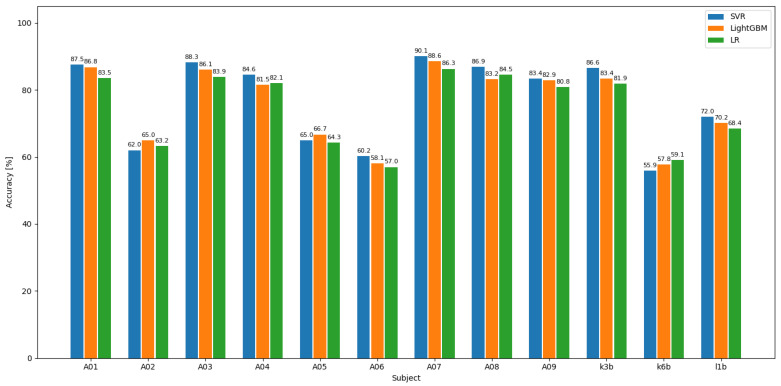
Comparison of accuracy using different models.

To assess the performance of the proposed feature extraction algorithm when applied to different classifiers, we used three classification models: Logistic Regression (LR) [37], LightGBM (Light Gradient Boosting Machine) [38] based on ensemble learning, and Support Vector Regression (SVR) to classify features. While Logistic Regression demonstrates superior performance for certain subjects and LightGBM shows advantages in addressing nonlinear boundaries, the SVR model outperforms the others in terms of classification accuracy, achieving the highest average accuracy across most subjects. The comparison results can be found in Figure 5.

We also evaluated the accuracy of four categories. The experimental results show the following accuracies for the four categories as shown in Table 8: 87.2% for the left hand, 83.1% for the right hand, 73.2% for the legs, and 64% for the tongue. These results show that the motor imagery accuracy for the left and right hands is relatively high, likely because these movements generate more distinct EEG signals, making them easier to distinguish. The lower accuracy for the legs and tongue suggests that these movement signals are weaker and more susceptible to noise and artifacts, resulting in poorer classification results. Overall, the classification of hand movements is relatively good, while the classification accuracy for the legs and tongue is relatively low, likely due to the distinguishability of EEG signals.

### 3.3. ODFC Model Evaluation

To evaluate the effectiveness of the proposed scene recognition method based on structured features derived from object detection, we conduct a series of experiments on a constructed dataset and compare different combinations of features. The experiments focus on four types of interpretable and lightweight features: (1) object presence (binary indicator), (2) object occurrence counts, (3) average detection confidence, and (4) grid-based confidence statistics.

Principal Component Analysis (PCA) is employed to compress the concatenated feature vectors, reducing redundancy and enhancing computational efficiency. The feature dimension is reduced to 50 while preserving more than 95% of the cumulative variance. For classification, a Random Forest classifier is applied with 100 trees, and the maximum depth of each tree is optimized using 10 cross-validation.

The classification performance under different feature combinations is summarized in Table 9.

The results clearly demonstrate that enriching the feature representation leads to consistent improvements in classification performance. The basic features (presence + count) already capture essential object-level information, achieving over 75% accuracy. The inclusion of average confidence enhances the semantic strength of category-level features. Most notably, integrating the grid-based confidence statistics significantly boosts spatial structure awareness, leading to the best performance with 92.2% accuracy and 92% F1 score. Despite dimensionality reduction via PCA, the model retains high discriminative power, indicating strong feature compactness and robustness.

Table 10 shows the accuracy for three scenes. These results show that the kitchen and living room have higher accuracy, indicating that the environmental features in these two scenes in the dataset are easier for the model to recognize and distinguish. The bedroom has lower accuracy, possibly due to the simpler environment or the lack of a bed in the image, which creates more distractions. Overall, the classification results for all three scenes are good, demonstrating the model’s stability across different scenarios.

Overall, these results validate that the proposed structured feature representation—especially when incorporating spatial distribution—effectively improves indoor scene classification and is well-suited for real-time deployment in robotic applications.

To further evaluate the performance of the proposed structured scene representation, we contrast our method with two representative end-to-end convolutional neural networks that are widely used in image classification tasks: ResNet18 [39] and MobileNetV3 [40]. Both models are initialized with ImageNet-pretrained weights and fine-tuned on our constructed dataset comprising three indoor scene categories: kitchen, living room, and bedroom. All methods are evaluated on the same dataset split (70% training, 30% testing) using Accuracy and F1 score as the evaluation metrics.

**Figure 6:**
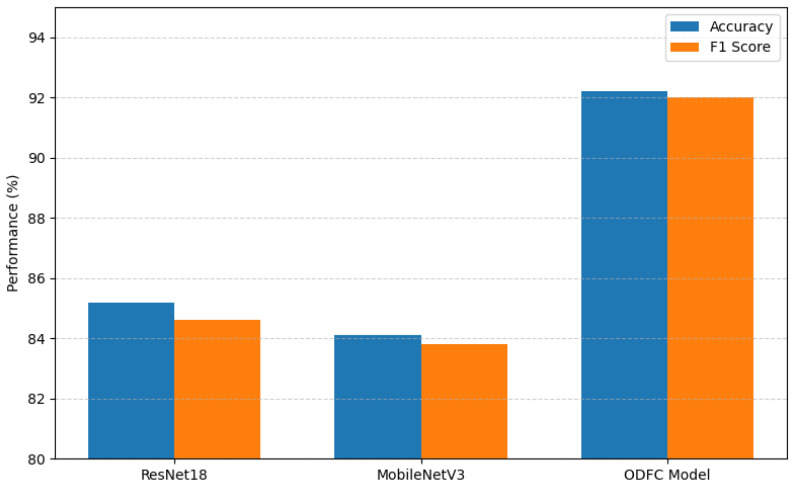
Comparison of scene classification methods.

Figure 6 demonstrates that our proposed method exceeds the performance of both ResNet18 and MobileNetV3 in terms of classification accuracy and F1 score. While CNNs can learn powerful representations, they typically require substantial computational resources and lack interpretability. In contrast, our method explicitly incorporates semantic object-level and spatial layout information, enabling more interpretable and efficient scene classification. This characteristic makes it ideal for deployment on platforms with limited resources, including mobile robots and embedded systems.

### 3.4. Evaluation of Multimodal Human–Robot Interaction Model

In order to verify the effectiveness of the joint reasoning based on EEG motor imagery and scene perception, this paper designed a task recognition experiment that integrates decision-level information. In the experiment, two sub-models were first trained separately: one for recognizing motor imagery EEG signals and the other for recognizing home scenes. In the reasoning stage, the Softmax probabilities output by the two models were fused, and the joint probability distribution of 12 types of robot tasks was constructed by multiplying the scene probability and the EEG category probability. Finally, the task with the maximum probability was used as the prediction result. The experiment evaluated the recognition performance of this method for 12 types of home robot tasks, and Table 11 presents the specific results. Overall, this method showed a high recognition accuracy in all task types, with an average classification accuracy of 83.4%, indicating that this method can effectively integrate multimodal information and realize intention-driven intelligent task recognition for the elderly.

## 4. Discussion

This paper introduces a multimodal human–robot interaction method that integrates EEG motor imagery and visual scene information, and applies it to the task of identifying and responding to the intentions of the elderly by household robots. The experimental results reveal that this method performs well in recognizing 12 typical household tasks, with an average accuracy of 83.4%, highlighting its strong practical value and potential for broader application.

From the results of task recognition, tasks such as KR-Deliver (deliver bowls and chopsticks for meal preparation), LL-Turn on (turn on TV or play music for leisure) and LR-Pour (pour and serve water or tea to the user) have obvious EEG pattern characteristics and scene differences, and the recognition accuracy is generally high (>85%), indicating that this method can effectively capture the cross-information of movement intentions and scene semantics. In scenarios such as BL-WakeSupport (assist the user in getting out of bed safely) and BR-MedicationAssist (deliver medicine and remind for medication schedule), the task recognition accuracy is relatively low. This may be influenced by the significant individual differences in EEG signals. Similar research findings have also appeared in the studies conducted by Kamrud et al., who pointed out that significant individual differences among subjects can affect the classification results of EEG signals [41].

The decision-level fusion strategy adopted in this paper has several advantages in multimodal perception tasks: on the one hand, this strategy achieves information fusion without increasing the complexity of the model by independently training the EEG classifier and the scene recognition model, and has good flexibility and scalability; on the other hand, the Softmax probability product fusion improves the robustness of the overall prediction while retaining the confidence information of each modality, which is particularly suitable for the independent update of sub-modules in actual system deployment. According to research by Yadav et al., the decision-level fusion method performs relatively well in multimodal tasks, as it can effectively handle heterogeneous information while maintaining high stability in dynamic environments [42].

However, current approaches also have limitations. For example, system stability under dynamic switching between multiple scenes, low-quality EEG acquisition, or scene blur requires further improvement. Similar challenges have been addressed in a study by W et al., where low-quality signals and environmental uncertainty can significantly degrade the performance of multimodal systems [43]. Furthermore, fusion methods do not consider temporal dependencies between modalities or introduce high-level semantic guidance mechanisms. Furthermore, our current study reduces the number of EEG channels to 15, leaving some distance to the realization of portable EEG signal acquisition. Future research could consider introducing temporal consistency constraints, attention-guided fusion mechanisms, or coincidence prediction methods to further enhance generalization and reliability in complex interactive environments. Our goal is to optimize the number of channels to less than ten, refine the entire human–robot interaction system through end-to-end experiments, and further optimize the system’s command response and feedback latency. Our previous research has conducted preliminary experiments on these issues, achieving certain results and providing valuable reference for future system optimization. As research continues [44], we hope to further improve the efficiency, robustness, and practicality of the system, opening up broader prospects for multimodal human-computer interaction applications.

## 5. Conclusions

This study improved the capabilities of user intention recognition and scene understanding by fusing EEG signals with visual scene information, overcame some limitations of traditional single-modal methods, and achieved the research goal of building a multimodal task recognition system for home service robots. Experimental results show that the proposed EEG channel selection and feature fusion strategy can maintain high-accuracy motor imagery classification while reducing data dimensions; at the same time, the YOLO v8 model also shows good performance in target detection and scene recognition tasks in home environments. Therefore, this multimodal method shows strong comprehensive capabilities in both intention perception and environmental understanding.

To enhance the system’s practicality and portability, this study adopts a channel screening strategy in the process of EEG signal acquisition and processing, retaining only limited key channels, which greatly improves the feasibility of system deployment while ensuring recognition performance. At the same time, in order to build a task execution mechanism, we designed an “intention-scene-task” mapping model, applied the recognition results to the task reasoning of home service robots, and realized automated decision-making based on user intention and scene information.

Finally, we designed and constructed a multimodal task response feedback mechanism based on the existing model. This mechanism can receive EEG and image input in real time, call the aforementioned model to complete intent recognition and scene classification, and generate response tasks based on the recognition results. In this feedback mechanism, we further verified the response effect and system stability of the system in a variety of typical scenarios.

This research supports the further development of multimodal fusion techniques in human–robot interaction and assistive services, providing foundational theories and technical approaches for collaborative systems that integrate EEG and visual perception. It also provides reference and reference for subsequent research in the direction of wearable BCI systems and real-time task scheduling mechanisms.

## Figures and Tables

**Table 1:** Command mapping.

Scene	Motor Imagery	Task Description	Task Name
Kitchen	Left hand	Start kitchen appliances for cooking	KL-Cooking
Kitchen	Right hand	Deliver bowls and chopsticks for meal preparation	KR-Deliver
Kitchen	Legs	Assist with cleaning or reaching the sink area	KG-Cleaning
Kitchen	Tongue	Check food temperature to ensure it is safe to eat	KT-Check
Living Room	Left hand	Turn on TV or play music for leisure	LL- Turn on
Living Room	Right hand	Pour and serve water or tea to the user	LR-Pour
Living Room	Legs	Provide mobility support for standing or walking	LG-Mobility
Living Room	Tongue	Remind or deliver snacks or fruit	LT-deliver
Bedroom	Left hand	Turn off lights for sleeping	BL-Switch
Bedroom	Right hand	Deliver medicine and remind for medication schedule	BR-MedicationAssist
Bedroom	Legs	Assist the user in getting out of bed safely	BL-WakeSupport
Bedroom	Tongue	Offer water or activate humidifier for hydration	BT-ServeWater

**Table 2:** Average accuracy under different filter frequency ranges.

Filter Range (Hz)	Accuracy (%)
4–40	73.2
8–32	74.5
8–30	73.8

**Table 3:** Average accuracy under different wavelet packet decomposition layers.

Decomposition Layers	Accuracy (%)
3 layers	74.5
4 layers	75.2
5 layers	76.8

**Table 4:** Average accuracy under different time window sizes.

Window Length	Accuracy (%)
1s	72.9
2s	76.8
3s	77.3

**Table 5:** BCI Competition IV Dataset 2a candidate channel selection results.

Subject	Channel (Score)												
A01T	**P1**	**CP3**	**Pz**	**POz**	**P2**	**CP1**	**CPz**	**CP4**	**CP2**	**FCz**	**C5**	**FC1**	Fz
	0.906	0.874	0.801	0.793	0.728	0.648	0.614	0.610	0.543	0.500	0.464	0.460	0.449
A02T	**FC2**	**C6**	**FC4**	**FC3**	**Fz**	**C2**	**CP4**	**FCz**	**FC1**	**CP2**	**C5**	**C1**	CPz
	0.831	0.824	0.790	0.684	0.682	0.617	0.580	0.566	0.553	0.539	0.500	0.470	0.466
A03T	**FC3**	**Pz**	**CPz**	**P2**	**FC4**	**POz**	**P1**	**CP1**	**CP2**	**FC1**	**CP4**	**Fz**	C1
	0.614	0.564	0.544	0.535	0.511	0.487	0.467	0.436	0.429	0.410	0.402	0.397	0.374
A04T	**CP4**	**C2**	**C6**	**FC4**	**CP2**	**P2**	**POz**	**Pz**	**CPz**	**FC2**	**FCz**	**P1**	CP1
	0.685	0.620	0.617	0.571	0.543	0.537	0.527	0.484	0.421	0.415	0.390	0.371	0.291
A05T	**Fz**	**FC3**	**FCz**	**FC1**	**FC4**	**FC2**	**P2**	**C1**	**POz**	**C2**	**CPz**	**CP4**	Pz
	0.805	0.756	0.748	0.721	0.506	0.501	0.376	0.370	0.351	0.344	0.332	0.308	0.281
A06T	**CP4**	**CPz**	**P1**	**Pz**	**P2**	**CP1**	**POz**	**CP2**	**Fz**	**FCz**	**C2**	**FC2**	CP3
	0.797	0.748	0.726	0.682	0.586	0.577	0.546	0.540	0.540	0.500	0.435	0.420	0.405
A07T	**CPz**	**C1**	**Pz**	**CP1**	**FCz**	**POz**	**P1**	**P2**	**C2**	**FC2**	**CP3**	**CP2**	FC1
	0.908	0.847	0.826	0.780	0.765	0.758	0.752	0.742	0.710	0.709	0.704	0.668	0.667
A08T	**CP4**	**C6**	**CP2**	**P2**	**C2**	**FC4**	**Pz**	**CPz**	**POz**	**FC2**	**FCz**	**P1**	Fz
	0.931	0.813	0.784	0.710	0.552	0.547	0.538	0.531	0.476	0.456	0.433	0.375	0.362
A09T	**CP4**	**FC4**	**POz**	**C6**	**FC3**	**P2**	**FC2**	**Pz**	**P1**	**Fz**	**CP2**	**FCz**	CP3
	0.716	0.596	0.511	0.504	0.407	0.407	0.385	0.368	0.335	0.319	0.317	0.279	0.273

**Table 6:** BCI Competition III Dataset 3a candidate channel selection results.

Subject	Channel (Score)												
k3b	**PO3**	**PO4**	**P6**	**TP8**	**FC3**	**FT7**	**P8**	**FC5**	**O2**	**F6**	**C6**	**P2**	CP4
	0.783	0.752	0.749	0.741	0.735	0.719	0.718	0.712	0.693	0.679	0.679	0.661	0.651
k6b	**FC2**	**FC1**	**P3**	**P8**	**P7**	**F8**	**AF7**	**P2**	**T8**	**FC6**	**F7**	**F3**	Pz
	0.670	0.633	0.631	0.619	0.613	0.602	0.593	0.590	0.585	0.580	0.564	0.559	0.554
l1b	**P6**	**P2**	**TP8**	**CP4**	**Oz**	**CPz**	**P5**	**C1**	**C5**	**C2**	**P1**	**C6**	F6
	0.950	0.883	0.751	0.745	0.736	0.701	0.653	0.592	0.581	0.567	0.562	0.540	0.488

**Table 7:** Classification accuracy (%) comparison across channel configurations.

Subject	ALL Channel	C3 C4 Cz	CSFF-Channel Selection
A01T	90.0	70.4	87.5
A02T	60.0	55.7	62.0
A03T	91.5	72.1	88.3
A04T	88.0	69.0	84.6
A05T	66.5	62.5	65.0
A06T	63.5	59.6	60.2
A07T	92.5	73.2	90.1
A08T	89.8	71.4	86.9
A09T	85.0	69.7	83.4
K3b	89.4	72.3	86.6
K6b	60.8	51.7	55.9
L1b	76.5	64.1	72.0

**Table 8:** EEG accuracy of each category.

Motor Imagery Task	Accuracy (%)
Left Hand	87.2
Right Hand	83.1
Legs	73.2
Tongue	64.0

**Table 9:** Scene classification performance under different feature combinations.

Feature Combination	Dimensionality	Accuracy	F1 Score
Presence + Count	160	75.1%	70.5%
Presence + Count + Confidence	240	85.4%	84.7%
Presence + Count + Confidence + Grid (PCA)	50	**92.2%**	**92%**

**Table 10:** The Accuracy of Each Scene.

Scene	Accuracy (%)
Kitchen	94.2
Living Room	93.3
Bedroom	89.1

**Table 11:** Task-wise accuracy using decision-level fusion (Softmax product).

Task Name	Scene	Motor Imagery	Accuracy (%)
KR-Deliver	Kitchen	Left Hand	86.5
KL-Cooking	Kitchen	Right Hand	84.3
KG-Cleaning	Kitchen	Legs	81.2
KT-Check	Kitchen	Tongue	85.1
LL-Turn on	Living Room	Left Hand	87.0
LR-Pour	Living Room	Right Hand	85.8
LG-Mobility	Living Room	Legs	82.4
LT-deliver	Living Room	Tongue	84.9
BL-Switch	Bedroom	Left Hand	83.7
BR-MedicationAssist	Bedroom	Right Hand	81.0
BL-WakeSupport	Bedroom	Legs	80.2
BT-ServeWater	Bedroom	Tongue	83.0
Average Accuracy	–	–	83.4

## Data Availability

A publicly available dataset was analyzed in this study. The data can be found here: https://www.bbci.de/competition/iv/ (accessed on 12 December 2008), https://www.bbci.de/competition/iii/ (accessed on 16 June 2005), https://cocodataset.org/ (accessed on 6 September 2014), http://places.csail.mit.edu/index.html (accessed on 1 July 2017).

## References

[1] Wolpaw J.R., Birbaumer N., McFarland D.J., Pfurtscheller G., Vaughan T.M. (2002). Brain–Computer Interfaces for Communication and Control. Clin. Neurophysiol..

[2] Kim H., Yoshimura N., Koike Y. (2019). Classification of movement intention using independent components of premovement EEG. Front. Hum. Neurosci..

[3] Kang H., Nam Y., Choi S. (2009). Composite Common Spatial Pattern for Subject-to-Subject Transfer. IEEE Signal Process. Lett..

[4] Ang K.K., Chin Z.Y., Zhang H., Guan C. (2012). Filter Bank Common Spatial Pattern Algorithm on BCI Competition IV Datasets 2a and 2b. Front. Neurosci..

[5] Jahankhani P., Kodogiannis V., Revett K. EEG signal classification using wavelet feature extraction and neural networks. Proceedings of the IEEE John Vincent Atanasoff 2006 International Symposium on Modern Computing (JVA’06).

[6] Yuan H., Liu T., Szarkowski R., Rios C., Ashe J., He B. (2010). Negative Covariation between Task-related Responses in Alpha/Beta-band Activity and BOLD in Human Sensorimotor Cortex. NeuroImage.

[7] Luo Y., Mu W., Wang L., Wang J., Wang P., Gan Z., Zhang L., Kang X. (2024). An EEG channel selection method for motor imagery based on Fisher score and local optimization. J. Neural Eng..

[8] Wang Z.M., Hu S.Y., Song H. (2019). Channel selection method for EEG emotion recognition using normalized mutual information. IEEE Access.

[9] Jiang X., Bian G., Tian Z. (2019). Removal of Artifacts from EEG Signals: A Review. Sensors.

[10] Jocher G., Chaurasia A., Stoken A. Ultralytics YOLOv8, 2023. GitHub Repository. https://github.com/ultralytics/ultralytics.

[11] Laulkar C., Kulkarni P. (2020). Integrated yolo based object detection for semantic outdoor natural scene classification. Applied Computer Vision and Image Processing, Proceedings of the ICCET 2020, Nanded, India, 9–11 January 2020.

[12] Zhang X., Du S., Zhang Y. (2018). Semantic and spatial co-occurrence analysis on object pairs for urban scene classification. IEEE J. Sel. Top. Appl. Earth Obs. Remote Sens..

[13] Krizhevsky A., Sutskever I., Hinton G.E. (2017). ImageNet Classification with Deep Convolutional Neural Networks. Commun. ACM.

[14] Simonyan K., Zisserman A. (2015). Very Deep Convolutional Networks for Large-Scale Image Recognition. arXiv.

[15] Dosovitskiy A., Beyer L., Kolesnikov A., Weissenborn D., Zhai X., Unterthiner T., Dehghani M., Minderer M., Heigold G., Gelly S. (2021). An Image is Worth 16 × 16 Words: Transformers for Image Recognition at Scale. arXiv.

[16] Zhu X., Liu J., Liu W., Ge J., Liu B., Cao J. Scene-aware label graph learning for multi-label image classification. Proceedings of the IEEE/CVF International Conference on Computer Vision.

[17] Zhang Y., Wang S., Sun P., Phillips P. (2015). Pathological brain detection based on wavelet entropy and Hu moment invariants. Bio-Med. Mater. Eng..

[18] Min J., Cai M., Gou C., Xiong C., Yao X. (2023). Fusion of forehead EEG with machine vision for real-time fatigue detection in an automatic processing pipeline. Neural Comput. Appl..

[19] Younis E.M., Zaki S.M., Kanjo E., Houssein E.H. (2022). Evaluating ensemble learning methods for multi-modal emotion recognition using sensor data fusion. Sensors.

[20] Liu X., Xu Z., Huang K. (2023). Multimodal emotion recognition based on cascaded multichannel and hierarchical fusion. Comput. Intell. Neurosci..

[21] Yang M.Y., Rosenhahn B., Murino V. (2019). Multimodal Scene Understanding: Algorithms, Applications and Deep Learning.

[22] Duan J., Zhuang L., Zhang Q., Zhou Y., Qin J. (2024). Multimodal perception-fusion-control and human–robot collaboration in manufacturing: A review. Int. J. Adv. Manuf. Technol..

[23] Choi D.Y., Kim D.H., Song B.C. (2020). Multimodal attention network for continuous-time emotion recognition using video and EEG signals. IEEE Access.

[24] Senior H., Slabaugh G., Yuan S., Rossi L. (2025). Graph neural networks in vision-language image understanding: A survey. Vis. Comput..

[25] Didolkar A., Zadaianchuk A., Goyal A., Mozer M., Bengio Y., Martius G., Seitzer M. (2024). Zero-shot object-centric representation learning. arXiv.

[26] Zhou B., Lapedriza A., Khosla A., Oliva A., Torralba A. (2018). Places: A 10 Million Image Database for Scene Recognition. IEEE Trans. Pattern Anal. Mach. Intell..

[27] https://www.bbci.de/competition/iv/.

[28] https://www.bbci.de/competition/iii/.

[29] St L., Wold S. (1989). Analysis of variance (ANOVA). Chemom. Intell. Lab. Syst..

[30] Ang K.K., Chin Z.Y., Zhang H., Guan C. Filter bank common spatial pattern (FBCSP) in brain-computer interface. Proceedings of the 2008 IEEE International Joint Conference on Neural Networks (IEEE World Congress on Computational Intelligence).

[31] Wu T., Yan G., Yang B., Sun H. (2008). EEG feature extraction based on wavelet packet decomposition for brain computer interface. Measurement.

[32] https://cocodataset.org/.

[33] Woodworth B., Patel K.K., Stich S., Dai Z., Bullins B., Mcmahan B., Shamir O., Srebro N. Is local SGD better than minibatch SGD?. Proceedings of the International Conference on Machine Learning PMLR.

[34] Crasto N. (2024). Class imbalance in object detection: An experimental diagnosis and study of mitigation strategies. arXiv.

[35] http://places.csail.mit.edu/index.html.

[36] Acharya J.N., Hani A.J., Cheek J., Thirumala P., Tsuchida T.N. (2016). American clinical neurophysiology society guideline 2: Guidelines for standard electrode position nomenclature. Neurodiagn. J..

[37] Zhou Y., Zeng C., Mu Z. (2023). Optimal Feature-Algorithm Combination Research for EEG Fatigue Driving Detection Based on Functional Brain Network. IET Biom..

[38] Ke G., Meng Q., Finley T., Wang T., Chen W., Ma W., Ye Q., Liu T.Y. (2017). Lightgbm: A highly efficient gradient boosting decision tree. Adv. Neural Inf. Process. Syst..

[39] He K., Zhang X., Ren S., Sun J. Deep residual learning for image recognition. Proceedings of the IEEE Conference on Computer Vision and Pattern Recognition.

[40] Howard A., Sandler M., Chu G., Chen L.C., Chen B., Tan M., Wang W., Zhu Y., Pang R., Vasudevan V. Searching for mobilenetv3. Proceedings of the IEEE/CVF International Conference on Computer Vision.

[41] Kamrud A., Borghetti B., Schubert Kabban C. (2021). The effects of individual differences, non-stationarity, and the importance of data partitioning decisions for training and testing of EEG cross-participant models. Sensors.

[42] Yadav S.K., Rafiqi M., Gummana E.P., Tiwari K., Pandey H.M., Akbara S.A. (2023). A novel two stream decision level fusion of vision and inertial sensors data for automatic multimodal human activity recognition system. arXiv.

[43] Grosselin F., Navarro-Sune X., Vozzi A., Pandremmenou K., de Vico Fallani F., Attal Y., Chavez M. (2019). Quality assessment of single-channel EEG for wearable devices. Sensors.

[44] Zhou T.H., Yang C., Wang L., Li D. Emotional Interaction Activities for Home Robots based on EEG Emotion Recognition. Proceedings of the 2024 IEEE International Conference on Medical Artificial Intelligence (MedAI).

